# Exploring of Primate Models of Tick-Borne Flaviviruses Infection for Evaluation of Vaccines and Drugs Efficacy

**DOI:** 10.1371/journal.pone.0061094

**Published:** 2013-04-09

**Authors:** Natalia S. Pripuzova, Larissa V. Gmyl, Lidiya Iu. Romanova, Natalia V. Tereshkina, Yulia V. Rogova, Liubov L. Terekhina, Liubov I. Kozlovskaya, Mikhail F. Vorovitch, Karina G. Grishina, Andrey V. Timofeev, Galina G. Karganova

**Affiliations:** FSBI Chumakov Institute of Poliomyelitis and Viral Encephalitides (IPVE) RAMS, Moscow, Russia; International Centre for Genetic Engineering and Biotechnology, Italy

## Abstract

Tick-borne encephalitis virus (TBEV) is one of the most prevalent and medically important tick-borne arboviruses in Eurasia. There are overlapping foci of two flaviviruses: TBEV and Omsk hemorrhagic fever virus (OHFV) in Russia. Inactivated vaccines exist only against TBE. There are no antiviral drugs for treatment of both diseases. Optimal animal models are necessary to study efficacy of novel vaccines and treatment preparations against TBE and relative flaviviruses. The models for TBE and OHF using subcutaneous inoculation were tested in *Cercopithecus aethiops* and *Macaca fascicularis* monkeys with or without prior immunization with inactivated TBE vaccine. No visible clinical signs or severe pathomorphological lesions were observed in any monkey infected with TBEV or OHFV. *C. aethiops* challenged with OHFV showed massive hemolytic syndrome and thrombocytopenia. Infectious virus or viral RNA was revealed in visceral organs and CNS of *C. aethiops* infected with both viruses; however, viremia was low. Inactivated TBE vaccines induced high antibody titers against both viruses and expressed booster after challenge. The protective efficacy against TBE was shown by the absence of virus in spleen, lymph nodes and CNS of immunized animals after challenge. Despite the absence of expressed hemolytic syndrome in immunized *C. aethiops* TBE vaccine did not prevent the reproduction of OHFV in CNS and visceral organs. Subcutaneous inoculation of *M. fascicularis* with two TBEV strains led to a febrile disease with well expressed viremia, fever, and virus reproduction in spleen, lymph nodes and CNS. The optimal terms for estimation of the viral titers in CNS were defined as 8–16 days post infection. We characterized two animal models similar to humans in their susceptibility to tick-borne flaviviruses and found the most optimal scheme for evaluation of efficacy of preventive and therapeutic preparations. We also identified *M. fascicularis* to be more susceptible to TBEV than *C. aethiops*.

## Introduction

A group of tick-borne mammalian flaviviruses includes agents of serious human diseases, such as tick-borne encephalitis (TBE), Powassan encephalitis, Omsk hemorrhagic fever (OHF), Kyasanur forest disease (KFD) and also viruses that do not lead to disease in humans in nature, such as Langat virus (LGT) and Louping ill virus.

TBE morbidity has increased by nearly 40% in Europe between 1974 and 2004 [1], and by 10-fold in Russia between 1974 and 1996, and currently remains high [2]. According to the data from the Russian Federal Service, 3,094 and 3,524 TBE cases have been registered in Russia in 2010 and 2011, respectively (http://rospotrebnadzor.ru). TBE in humans can progress as inapparent or acute forms with varying degrees of severity. In Russia 3–10% of acute TBE cases become chronic. Morbidity of Powassan, OHF and KFD is sporadic [3]–[5] and there are no vaccines against them.

The only well-evaluated and effective preparation against TBE is inactivated concentrated purified vaccine. Currently, there are 4 commercial vaccines against TBE virus (TBEV) that demonstrate a good effectiveness [6]–[7]. Nevertheless, the necessity for revaccinations every 3–5 years, probability of allergic reactions in recipients, and rare registration of TBE cases among vaccine recipients [8]–[9] have necessitated the development of new approaches to design new prophylactic preparations. During recent years several studies have been conducted to develop new generation vaccines against TBE [10]–[20]. There are no drugs for emergency prophylaxis and treatment of TBE, although the research studies based on various approaches are being conducted [21]–[24]. So, the selection of methods for estimation of vaccine and drug safety and protective efficacy is highly critical.

Laboratory mice are a convenient and widely used model of TBE. Most laboratory mouse strains are more susceptible to TBE infection than humans, because even a very small dosage of the wild type virus (as low as 1 plaque forming unit (PFU)) is enough to cause severe illness and death [18]. However, inapparent forms of TBE in humans are much more frequent than clinical forms. This is confirmed by epidemiological data showing the number of seropositive people in endemic territories. Mice are not suitable for modeling sublethal and chronic forms of TBE. This can be important for the efficacy evaluation of antiviral preparations, because the immune response and effects of immunomodulators in various forms of infection can vary significantly. Therefore, although mice are more sensitive and an easier-to-handle model for TBEV, they do not recapitulate all aspects of human infection.

Traditionally, it has been considered that monkeys are not susceptible to peripheral inoculation of neurotropic flaviviruses [25]–[29]. However, several studies have shown that monkeys may have a clinical manifestation and pathomorphological (PM) lesions in CNS similar to humans after infection with different strains of TBE or Louping ill viruses [30]–[32]. Recently obtained data have shown that laboratory mice can be less susceptible than monkeys to a new chimeric attenuated flavivirus, which has been proposed as a vaccine candidate [18]. Susceptibility of the non-human primates to TBEV has also been demonstrated in the described case of TBE in monkey (*Macaca sylvanus*) after its natural exposure in the endemic area [33]. All these facts suggest that monkeys are close to humans by their susceptibility to tick-borne flavivirus infection and can be used as an alternative model that may complement the vaccine and drug testing in mice.

Two basic models for protective efficacy evaluation of vaccine candidates have been employed earlier in monkeys. The most frequently used approach is subcutaneous (s/c) challenge of immunized monkeys and estimation of viremia level in the first 7–10 days after challenge relative to non-immunized animals. Although many studies have shown that viremia after s/c inoculation is very low, even in non-immunized monkeys, it can depend on the monkey species and virus strain used [15], [34]–[36]. The second model is intracerebral (i/c) challenge with virulent strains of flaviviruses [35], [37]–[39]. However, this model is not very informative because s/c or peroral immunization does not provide complete protection against the direct inoculation of TBEV into CNS [35], [37]–[38].

In the current study we concentrated on the development of the most appropriate and informative model for estimation of protective efficacy of preventive and therapeutic preparations against TBEV in monkeys. We found that non-human primates, although not ideal, can be successfully used for vaccines and antiviral drugs testing. We modeled immunization with commercial inactivated vaccine against TBE and s/c challenge of two different monkey species with two highly virulent TBEV strains and one OHF virus (OHFV) strain. We compared the animals' susceptibility, TBEV strains' virulence, the terms of experiments and the target organs that could serve as the markers of propagation of the challenging virus for the estimation of protection level. Monkeys of the *Macaca* genus were found to be the most permissive model of TBEV for the protective efficacy evaluation.

## Materials and Methods

### Cells and Viruses

Pig embryo kidney (PEK) cells were received from the IPVE collection. This cell line was established in 1970s and is permissive to TBEV; it has been used previously to perform plaque titration assay [18], [40].

TBEV Absettarov strain (GenBank Accession # AF091005) was isolated from the blood of a patient in the Leningrad region of the USSR in 1951 and belongs to Western TBEV subtype [41]. TBEV SofjinKGG strain (GenBank Accession # GU121963) was isolated from human brain in the Khabarovsk region of the USSR in 1937 and belongs to Far-Eastern TBEV subtype [40]. Both TBEV strains were from the laboratory virus collection. OHFV Nikitina strain (GenBank Accession # GU290187), isolated from the blood of a patient in 1945, was received from the virus collection of Chumakov IPVE (kindly provided by Dr. Vanda V. Pogodina). The stocks of viruses were passaged in white mice and were stored at −70°C as 10% brain suspension of infected mice. To obtain the clones of two TBEV strains we used three sequential plaque clonings in PEK cells. The recovered clones (18A of Absettarov strain (Abs-18) and clone 16S of SofjinKGG (Sof-16) strain) were additionally passaged through PEK cells and characterized by plaque size and viremia level in mice after intraperitoneal (i/p) inoculation. Virulence for adult mice, plaque phenotype and titers in PEK cells, as well as biochemical properties of virions of these TBEV strains and its derivate clones have been characterized and described previously [40].

### Monkey Experiments

The study (including experiments in monkeys and mice) was approved by FSBI (Federal State Budgetary Institution) Chumakov Institute of Poliomyelitis and Viral Encephalitides (IPVE) RAMS (Russian Academy of Medical Sciences), (Moscow, Russia) ethics committee and conducted according to the institution's bioethical regulations of research conduct in humans and animals, dated December 25, 2008; and international guidelines for animal husbandry, including recommendations of CIOMS, 1985; FESLA Working Group Report, 1996–1997; the Weatherall report. African Green monkeys (*Cercopithecus aethiops*), 7 males and 6 females, weighting 2.1–4.9 kg were received from African Animals Ltd., Tanzania under CITES permit # 13869. Nine (9) males of Crab-eating macaques (*Macaca fascicularis*), weighting 3.7–5.0 kg were received from Bioculture (MTIUS) LTD (Mauritius) under CITES permit # MU 031286.

The animals were housed in individual cages 1 m×70 cm×80 cm in the same room, having 12 hours of day light and balanced feeding (fruits, vegetables, cereals, bread and vitamins daily). Special construction of cages allowed fast and easy fixation of an animal. Bleeding and temperature measurement were performed by qualified personnal for a minimum amount of time. A mixture of “Zooxylazinum” and “calypsol” was used as a light anaesthesia. At the end of the study all animals were sacrificed to perform histological and virological analysis of internal organs. Animals were euthanized by intramuscular injection with hexobarbital and the death was verified by respiratory and cardiac arrest and the absence of pupillary light reflex.

Sera of monkeys were free from antibodies against TBEV and OHFV in the plaques neutralization assay and had no adventitious agents pathogenic for 4 weeks-old mice after i/c inoculation. Randomized animals were inoculated s/c with 0.5 ml of vaccine or 0.5 ml of a challenging virus under the shoulder blade. For immunization of monkeys commercial lots of purified concentrated dry inactivated vaccines against TBE from the Federal State Unitary Enterprise of Chumakov IPVE (EIPVE) (Moscow, Russia) or “FSME-Immune inject” (Baxter, Austria) were used. Vaccines were administered twice in 0.5 ml with an interval of 29 days (EIPVE vaccine) or with an interval of 35 days (“FSME-Immune inject”) between vaccinations.

After the challenge with TBE or OHF viruses, animals were monitored twice a day for clinical signs until the end of experiment; rectal temperature (T) was measured daily for 2 weeks before and after infection. The upper limit of T (UL) for each monkey was calculated according to the formula (for 95% confidence): UL = Tm+1.96×SD/√n, where Tm is the mean normal T obtained from the data range before the experiment, SD - standard deviation, and n – the number of obtained T values. Fever was statistically significant if it was higher than UL.

Monkeys were bled daily for 8 days and on the 14^th^ day after infection; viremia levels in serum and in clot were tested by plaque assay. Sera were also used for antiviral antibody titration in ELISA and plaque reduction neutralization assay. Complete blood count and biochemical analysis were performed in the Center on molecular diagnostics FSBI VGNKI (Moscow). On various terms post infection animals were euthanized under Hexenalum anesthesia. Brain, spinal cord, lymph nodes, liver, kidney, spleen and lung were fixed in buffered 10% formalin for histological examination. Samples of the frontal lobe, cerebellum, spinal cord (dorsal cord and caudal part of lumbar cord) and samples of the visceral organs (approximately 0.5 cm^3^) were frozen at −70°C and used for virological tests.

### Plaque assay

Virus titers (of TBEV or OHFV) were determined by plaque assay in PEK cells as described earlier [40]. Virus titers were expressed as logarithm of plaque forming units per milliliter (log_10_PFU/ml) for virus infected cell culture supernatant, animal serum or 10% tissue (brain, liver, spleen, etc.) suspensions. In all cell culture based assays media and supplements produced in EIPVE (Moscow, Russia) and FBS produced in Furo (Moscow, Russia) were used.

### ELISA

For antiviral antibody (AB) titration ELISA was performed using standard technique by the following scheme: 1^st^ layer – antigen (AG), 2^nd^ layer – 2-fold dilutions of analyzed sera, 3^rd^ layer – anti-monkey antibodies. AG was prepared from concentrated PEK cell culture supernatant by the following protocol. Cells were infected with 10% brain suspension of mice infected with TBEV strains SofjinKGG or Absettarov or OHFV strain Nikitina. On the 2^nd^ or 3^rd^ day post infection with first signs of cytopathogenic effect (CPE) cell culture supernatant was collected, cleared from the cell debris by low speed centrifugation, and then concentrated using ultracentrifugation. The pellet was dissolved in PBS. The negative control AG was prepared from non-infected cells using the same method after one freeze and thaw cycle. Viral and negative control AGs were equilibrated by total protein containment. Results were registered with ELISA optical reader (Titertek Multiscan). The final AB titer was calculated as the last sera dilution that gave an optical signal with the viral AG twice higher than with non-infected cells control. All sera were tested in at least 2 replicates.

### Plaque reduction neutralization assay (prNA)

prNA was performed with TBEV Absettarov strain, TBEV SofjinKGG strain or with OHFV Nikitina strain in PEK cells using a standard technique. Briefly, serial two-fold sera dilutions in medium with 10% FBS were added to equal volumes of virus diluted to yield about 40 PFU/0.4 ml. Virus-serum dilution mixtures were incubated for 60 min at 37°C. Control dose of virus was incubated at the same time with 10% FBS medium. Three replicates of cells' monolayers in 6-well flasks were inoculated with 0.4 ml each of virus-serum mixtures and incubated for 60 min at 37°C. Then the monolayers were overlaid with agar as described for plaque assay. Final plaque counts were made in 4–5 days and results were calculated using the method of Reed and Muench [42].

### RT-PCR Analysis

Total RNA was extracted from 0.5 ml of 10% tissue suspensions using TRI Reagent LS (Sigma) according to the manufacturer's instructions. To ensure the quality of downstream steps 4 log_10_ PFU of Oral Poliovirus Vaccine type 3 (OPV3) was added to each tissue suspension as an internal control and the control PCR for OPV3 was performed as described previously [18], [43]. Reverse transcription (RT) was carried out with a random primer (Sintol, Russia) and M-Mlv recombinant reverse transcriptase (Promega) according to the manufacturer's protocol. PCR was performed in 25 µl using GeneAmp PCR System 2400 (Perkin Elmer). Detection of OHFV cDNA was carried out with primers FSM-1 (5′-GAGGCTGAACAACTGCACGA-3′) and FSM-2 (5′-GAACACGTCCATTCCTGATCT-3′) resulting in a product of 360 b.p. [44].

### Histological Examination

After 5–7 days of fixation in 10% formalin in PBS brain and visceral organs sections were made from the frontal surface of the tissue blocks as described earlier [18]. Then material was processed according to the standard technology for light microscopy. The resulting brain and spinal cord sections were stained by the Nissl method. Other organs' sections were stained with hematoxylin-eosin method. Blinded histological evaluation was done by a single investigator using the criteria for lesions scoring of the neurotropic viruses according to the established grading scale [45]–[46].

## Results

### 1. Design of the study

**Figure 1:**
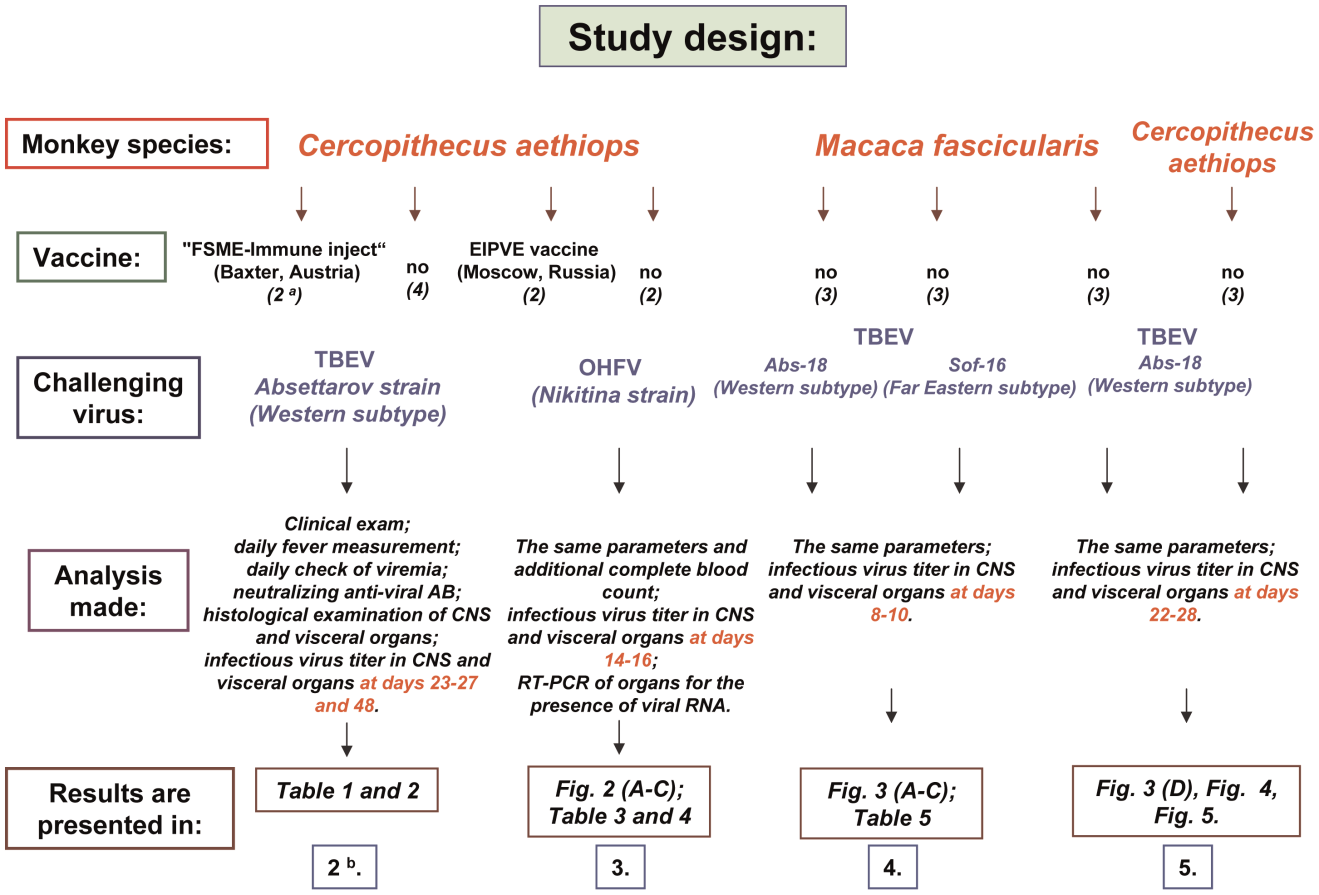
Study design. The flow chart represents the study design and performance. The monkey species used in each experiment is shown in red. Vaccine: the name of the vaccine administered before challenge or “no” vaccine is indicated. ^a^ - the number of monkeys used in each experiment is shown in parenthesis. ^b^ - the blue framed box represents the section number, which describes the results of each particular experiment. The challenging virus is shown in blue. “Analysis made”: the types of analysis and parameters used to evaluate the model are listed. “Results0 are presented in”: the results presented in figures or tables in the current study are listed.

The current study was designed and completed as shown in Figure 1. The first two experiments were performed in *C. aethiops* using s/c challenge with the Western subtype of TBEV (Absettarov strain) or OHFV (Nikitina strain) with or without prior vaccination with commercial inactivated vaccines. The next experiment was performed in *Macaca fascicularis* as a model for TBEV infection by s/c inoculation of two different strains after plaque cloning: Abs-18 (Absettarov strain, clone 18, Western subtype of TBEV) or Sof-16 (SofjinKGG strain, clone 16, Far Eastern subtype of TBEV). In the last experiment both monkeys' species were used in a parallel comparison of their sensitivity to TBEV; using the more virulent Abs-18 strain.

In all experiments the standardized analysis was performed after s/c challenge following the same scheme (defined in Figure 1): clinical examination; daily fever measurement; daily check of viremia; neutralizing anti-viral AB measurement; histological examination of CNS and visceral organs; infectious virus titer in CNS and visceral organs. The last 2 analyses were purposely made at the different (late or early) terms after infection, to see which of the terms are more appropriate to detect the infectious virus reproduction or patho-morphological lesions in the infected animals (the terms are highlighted in red in Figure 1). The number of animals used in each experiment is shown in parenthesis; the section numbers where the results are described are shown in the blue framed boxes. In addition, in Figure 1, we defined what figure or table represents the most significant results.

### 2. Evaluation of protective efficacy of the commercial inactivated vaccine against TBEV in *Cercopithecus aethiops* model

Two *C. aethiops* monkeys (#18 and 23) were twice s/c vaccinated with the commercial lot of inactivated vaccine against TBE (“FSME-Immun inject”, Baxter, Austria) with an interval of one month between vaccinations. In five weeks after the 2^nd^ vaccination both monkeys were s/c challenged with Absettarov strain in a dose of 6.7 log_10_ PFU. Four non-immunized monkeys (#11, 13, 19 and 22) also received the same dose of TBEV. To estimate the level of protection we used clinical examination, daily fever measurement, determination of infectious virus titer in blood, CNS and visceral organs, histological examination of CNS and visceral organs, and the anti-viral AB titers measured by prNA and ELISA.

No clinical signs after s/c virus inoculation were registered in any monkey except light fever in 3 out of 4 non-immunized animals: on the first day post-infection (d.p.i.) in monkey #19, on the 3^rd^ d.p.i. in monkey #22, on the 2^nd^ and 6^th^ d.p.i. in monkey #13. None of the immunized animals had fever.

Two immunized (#18 and 23) and two non-immunized animals (#19 and 22) were euthanized on the 23–27 d.p.i.; two non-immunized monkeys (#11 and 13) were euthanized on day 48. Histological analysis revealed slight morphological lesions in the CNS of non-immunized monkey #11 on the 48^th^ d.p.i. expressed in poorly defined degenerative changes of neurons (at score 1) in lumbar and cervical parts of spinal cord, cerebellum, medulla, middle brain and thalamus (the general average Nathanson score in CNS was 0.45). No histological lesions were found in the CNS of other animals. No specific changes were found in the liver, kidneys or heart of any animal. In some animals moderate macrophage reaction in marginal sinus of spleen, focal infiltration with macrophages and segmented leukocytes from peritoneum, hyperemia and proliferation of reticulo-endothelial elements in the organ stroma were observed. In the lymph nodes the expressed plethora and hyperplasia follicles with large reactive centers and marked macrophage reaction were noticed. Such changes are reactive and can be developed in response to the infection in an animal.

The level of viremia was very low even in non-immunized animals. We could reveal an infectious virus in sera only in two of the animals in very low titers: in monkey #11 on the 4^th^ d.p.i. at 1.5 log_10_ PFU/ml and in monkey #13 on 2^nd^ d.p.i. at 1.8 log_10_ PFU/ml. Therefore, viremia in this model (*C. aethiops*) could not be used as a measure of protection.

The CNS and visceral organs were analyzed for the presence of infectious virus. No infectious virus was revealed in any organ of monkeys #11 and 13 on the 48^th^ d.p.i. Virus was detected only in a low titer in a cortex of one non-immunized monkey (#22) on the 23^rd^ d.p.i. (Table 1). However, we detected virus in spleen and lymph nodes of non-immunized animals (#19 and 22) in rather high titers (2.7–4.4 log_10_ PFU/ml) at the same terms. No virus was revealed in any organs of immunized monkeys (Table 1). Thus, immunization with inactivated vaccine protected animals against reproduction or persistence of TBEV in organs of the immune system.

**Table 1:** Viral titers (log_10_PFU/ml) in CNS and visceral organs of *C. aethiops* monkeys, immunized twice with TBE inactivated vaccine (FSME-Immun), and in non-immunized monkeys on the 23–26 days after s/c challenge with 6.7 log_10_ PFU of TBEV, strain Absettarov.

	Non-immunized monkeys		Immunized monkeys	
Monkey ID	#19	#22	#18	#23
	**CNS**			
cortex	0	**2.0**	0	0
frontal lobe	0	0	0	0
cerebellum	0	0	0	0
thoracic part of a spinal cord	0	0	0	0
caudal part of a spinal cord	0	0	0	0
	**Visceral organs**			
kidney	0	0	0	0
liver	0	0	0	0
lung	0	0	0	0
spleen	**3.5**	**4.4**	0	0
submaxillary lymph nodes	**2.7**	**3.9**	0	0
axillary lymph nodes	**3.6**	**3.6**	0	0

0–<1 PFU in 0.1 ml of 10% suspension.

Both immunized monkeys demonstrated the presence of neutralizing AB against TBEV after immunization with inactivated vaccine. In a month after the 1^st^ immunization titers of neutralizing AB in sera of monkeys #18 and #23 were 1∶32 and 1∶64, correspondingly, and in a month after the 2^nd^ immunization - 1∶32 and 1∶512. To estimate the booster after challenge with TBEV we analyzed the dynamics of antiviral AB titers against TBEV in sera of monkeys on the early terms after challenge by ELISA (Table 2). Antiviral AB titers increased already on the 1^st^ d.p.i. in both immunized animals up to 1∶4,000. A little decrease (in double) in AB titers on the 2^nd^ d.p.i. was registered in immunized monkeys probably due to binding of AB to the newly produced virus.

**Table 2:** The average reciprocal antivirus antibody titers (mean for two monkeys) measured by ELISA in sera of immunized and non-immunized *C. aethiops* monkeys before and after challenge with 6.7 log_10_ PFU of TBEV, Absettarov strain.

Status	Days after challenge					
	0	1	2	5	8	21
No vaccine (2 monkeys)	0	0	0	0	**40**	**160**
Inactivated vaccine twice (2 monkeys)	**128**	**4,096**	**2,048**	**4,096**	**16,384**	**2,560**

### 3. Evaluation of efficacy of the commercial inactivated TBE vaccine against Omsk hemorrhagic fever virus (OHFV) in *Cercopithecus aethiops* model

Two *C. aethiops* monkeys (#2 and 4) were twice s/c vaccinated with the commercial lot of inactivated vaccine against TBE (EIPVE) with an interval of one month between vaccinations. In three weeks after the 2^nd^ vaccination both monkeys were s/c challenged with OHFV, strain Nikitina in a dose of 7.3 log_10_ PFU. Two non-vaccinated monkeys (#1 and 3) also received the same dose of OHFV. To estimate the disease progress and the level of protection we used clinical examination and histology, complete blood count and biochemical analysis of blood, determination of infectious virus titers in blood clots on the 2^nd^, 8^th^ and 14^th^ d.p.i., and in the CNS and visceral organs at days 14–16; RT-PCR analysis of organ suspensions for the presence of viral RNA, and prNA for the anti-viral AB titration.

**Figure 2:**
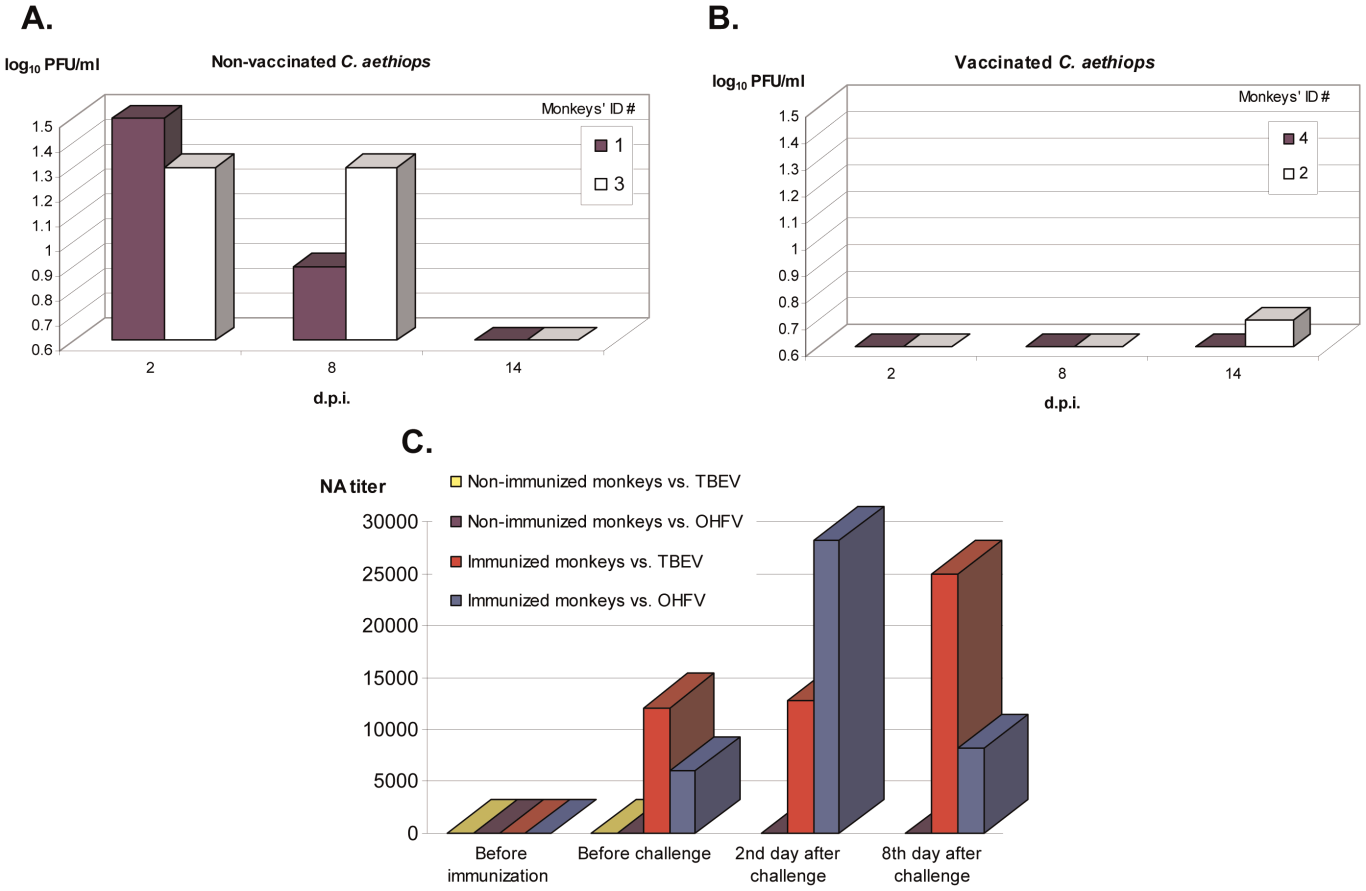
Viremia and neutralizing antibody titers in monkeys infected with 7.3 log_10_ PFU of OHFV. A. Viremia in two non-vaccinated monkeys (#1 and 3). B. Viremia in two monkeys (# 2 and 4), vaccinated with commercial inactivated vaccine against TBE (EIPVE). Viremia was analyzed in blood clots by plaque assay; the limit of detection of the plaque assay was 0.5 log_10_ PFU/ml. D.p.i. – days post-infection. C. Average (for two monkeys) reciprocal neutralizing antibody (NA) titers in sera of immunized and non-immunized *C. aethiops* monkeys before and after challenge. Two different viruses were used for neutralization assay: TBEV or OHFV.

No visible clinical signs of disease (neurological or hemorrhage related) were registered in any monkeys. On the 2^nd^ and 8^th^ d.p.i. infectious virus was registered in the blood of non-immunized animals (#1 and 3) (Figure 2A). On the 14^th^ d.p.i. a low titer of infectious virus in the blood was registered in one (#2) of two immunized monkeys (Figure 2B).

The complete blood count was performed in all monkeys before challenge and on the 2^nd^, 8^th^ and 14^th^ d.p.i. As shown in Table 3 on the 8^th^ d.p.i. non-immunized animal #3 had a massive hemolytic syndrome (hypochromic anemia, decrease of hemoglobin level in more than 50%), thrombocytopenic purpura (decrease of platelets in more than 50%), leukocytopenia, and 3-fold decrease of hematocrit value. On the 14^th^ d.p.i. we observed a slight change in all described indicators towards the normal level. The level of aspartate aminotransferase (AST) activity almost doubled on the 2^nd^ d.p.i. followed by normalization on the 14^th^ d.p.i. against the background of normal activity count of alanine aminotransferase (ALT) that probably caused by massive hemolysis. Unfortunately, we did not obtain data for non-vaccinated animal #1 on day 8. Nevertheless, as shown in Table 3, this monkey has the same picture as monkey #3 with the exception of the leukocyte count, although we cannot exclude that this animal also had a leukocytopenia before day 14.

**Table 3:** The complete blood count in *C. aethiops* monkeys before and at the different terms after s/c challenge with 7.3 log_10_ PFU of OHFV strain Nikitina.

	Non-immunized monkeys						Immunized monkeys					
Days after challenge	0		8		14		0		8		14	
Monkey ID	#1	#3	#1	#3	#1	#3	#2	#4	#2	#4	#2	#4
leukocytes (10^9^/l)	***5.9***	**5.3**	ND	**1.8**	***5.7***	**2.6**	***6.0***	**6.3**	***7.4***	**7**	***6.0***	**8**
platelets (10^3^/l)	***360***	**345**	ND	**97**	***140***	**147**	***290***	**450**	***294***	**333**	***282***	**316**
hemoglobin (g/L)	***116***	**129**	ND	**59**	***63***	**68**	***98.1***	**122**	***118***	**115**	***113***	**92**
erythrocytes (10^12^/L)	***5.1***	**5.0**	ND	**2.73**	***2.92***	**3.36**	***4.6***	**5.7**	***4.47***	**5.15**	***4.76***	**4.08**
hematocrit %	***51***	**56**	ND	**18.5**	***19.5***	**22.3**	***47***	**51**	***36.7***	**37**	***35.1***	**28.3**
Days after challenge	**0**		**2**		**14**		**0**		**2**		**14**	
Monkey ID	#1	#3	#1	#3	#1	#3	#2	#4	#2	#4	#2	#4
AST (U/l)	ND	**59**	***98***	**112**	***78.7***	**60.2**	***30***	**35**	***65.9***	**57**	***58***	**36.8**
ALT (U/l)	ND	**71**	***106***	**57**	***106***	**82.5**	***35***	**58**	***48.3***	**38**	***102***	**45.8**

ND – not determined; aspartate aminotransferase (AST); alanine aminotransferase (ALT).

In contrast to non-immunized animals, both vaccinated monkeys (#2 and 4) had almost no hemolytic syndrome with stable count of erythrocytes, platelets and leukocytes (Table 3). Some increase in AST activity and decrease of hematocrit value were noticed but not as much as observed in non-immunized animals.

No hemorrhagic signs or PM lesions in the CNS or visceral organs were revealed in any of the monkeys on the 14^th^–16^th^ days after challenge (data not shown). The CNS and visceral organs were analyzed for the presence of infectious virus on the 14^th^–16^th^ d.p.i. and for the presence of viral RNA by RT-PCR analysis. Infectious virus and viral RNA were registered in organs of either vaccinated or non-immunized monkeys (Table 4). In non-immunized monkey #3 virus was registered in a frontal lobe (2.2 log_10_ PFU/ml of 10% suspension) and viral RNA was detected in cortex and cerebellum, at the same time in immunized monkey #2 infectious virus was registered in the thoracic part of the spinal cord (1.4 log_10_ PFU/ml). In two other monkeys no virus or viral RNA was registered. The infectious virus was revealed in non-immunized monkeys (#1 and 3) in the liver, spleen, lungs, and axillary lymph nodes in titers of 0.9–4.1 log_10_ PFU/ml; in immunized monkeys, virus was registered in lungs, kidneys, and axillary lymph nodes in titers of 1.7–2.0 log_10_ PFU/ml (Table 4).

**Table 4:** Virus titers (log_10_ PFU/ml) and the presence or absence of viral RNA (+/−) in CNS and visceral organs of *C. aethiops* monkeys immunized and non-immunized with inactivated vaccine against TBE (EIPVE) on the 14–16 days after challenge with 7.3 log_10_ PFU of OHFV, strain Nikitina.

	Non-immunized monkeys		Immunized monkeys	
Monkey ID	#1	#3	#2	#4
	**CNS**			
cortex	0/−	0/+	0/−	0/−
frontal lobe	0	**2.2**	0	0
cerebellum	0/−	0/+	0/−	0/−
thoracic part of a spinal cord	0	0	**1.4**	0
caudal part of a spinal cord	0/−	0/−	0/−	0
	**Visceral organs**			
lungs	**2.1**	**1.8**	**1.9**	0
liver	**1.1**	**1.0**	0	0
spleen	**4. l**	0/+	0/−	0/−
kidneys	0	**2.3**	**2.0**	0
submaxillary lymph nodes	0	0	0	0
axillary lymph nodes	**0.9**	0	0	**1.7**

0–<1 PFU in 0.4 ml 10% suspension.

“−”– RT-PCR is negative for viral RNA.

“+”– RT-PCR is positive for viral RNA.

PrNA, conducted before and after challenge with OHFV (Figure 2C), showed that the level of neutralizing AB to both TBEV and OHFV in sera of immunized animals was very high (>6,000) compare to non-immunized monkeys (<20). In addition, AB titers doubled already by the 2^nd^ day after challenge in both vaccinated monkeys.

### 4. TBE infection in *Macaca fascicularis* monkeys after s/c inoculation with different strains of TBE virus

The level of viremia can be governed by the monkey species or virus properties. The diversity in the original virus population could create high variability in different animals in terms of the rate of virus reproduction in blood, visceral organs and CNS. So, in the next experiment we used another model, *M. fascicularis*, and two different TBEV strains: Absettarov and SofjinKGG after additional plaque cloning. The recovered clones (18A of Absettarov strain (Abs-18) and clone 16S of SofjinKGG strain (Sof-16)) were selected based on the virulence for adult mice after i/p and i/c inoculation and viremia level in mice after i/p inoculation. Plaque phenotype and titers in PEK cells, as well as the biochemical properties of virions of the parental TBEV strains and its derivate clones have been also characterized and described [40].

**Figure 3:**
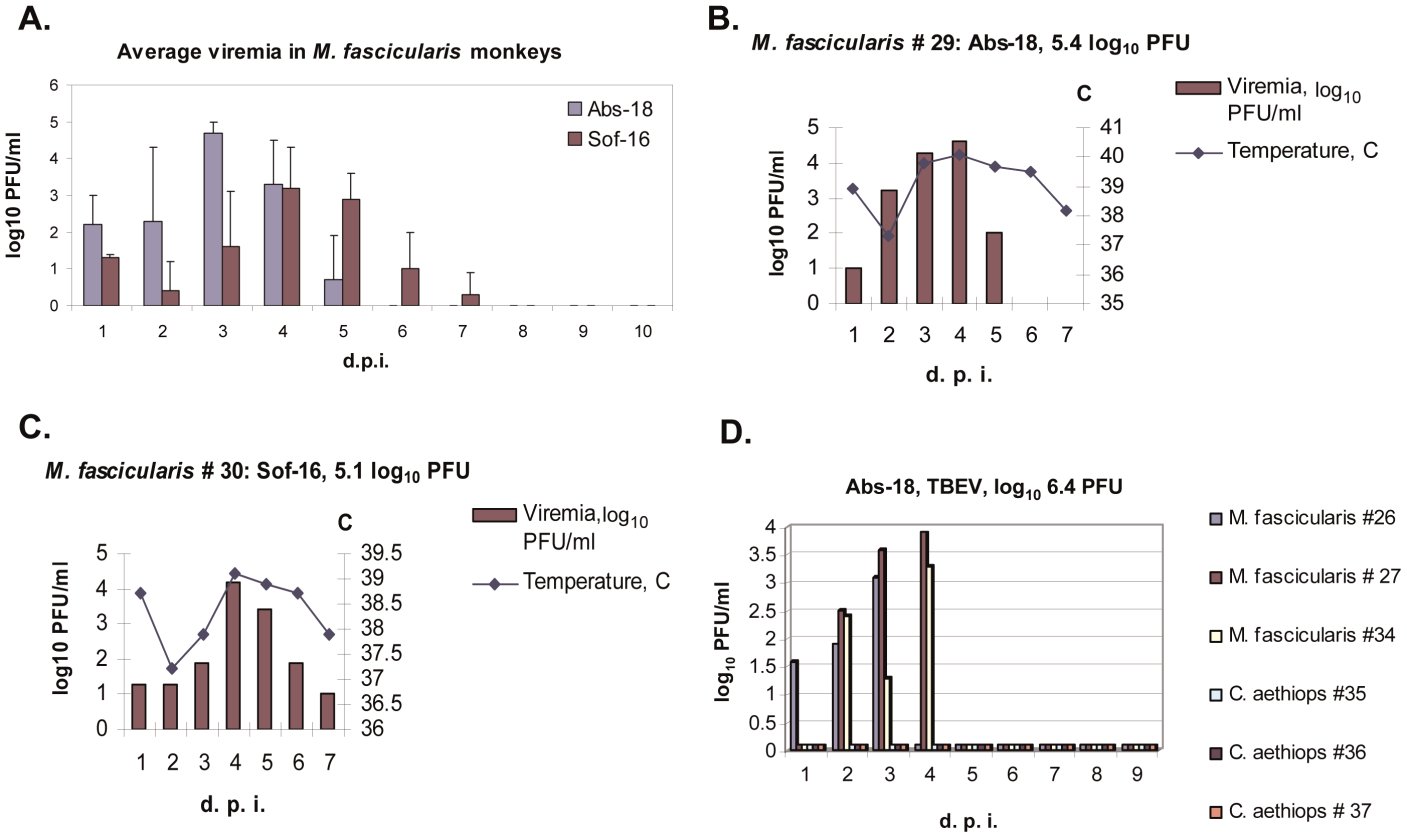
Analysis of viremia dynamics in different species of monkeys s/c infected with different TBEV strains. A. Dynamics of average viremia in *M. fascicularis* s/c infected with 5.4–6.4 log_10_ PFU of Abs-18 strain (monkeys #28, 29, 33), or with 5.1–6.1 log_10_ PFU of Sof-16 strain (monkeys #25, 30, 31). Viremia was analyzed in sera by plaque assay; the limit of detection of the assay: 0.7 log_10_ PFU/ml. The bars on the graph denote standard deviation calculated for three monkeys in each group for a giving d.p.i. B–C. Correlation of fever with dynamics of viremia in a serum of *M. fascicularis* (#29) infected with 5.4 log_10_ PFU of Abs-18 (B) and *M. fascicularis* (#30) infected with 5.1 log_10_ PFU of Sof-16 (C). D. Dynamics of viremia in sera of three *M. fascicularis* monkeys (#26, 27, 34) and three *C. aethiops* monkeys (#35, 36, 37) s/c infected with 6.4 log_10_ PFU of Abs-18. Viremia was analyzed by plaque assay; the limit of detection of the assay: 0.7 log_10_ PFU/ml. D.p.i. – days post-infection.

Three *M. fascicularis* monkeys (#28, 29, 33) were inoculated s/c with 5.4 and 6.4 log_10_ PFU of Abs-18 and 3 monkeys (#25, 30, 31) were inoculated with 5.1 and 6.1 log_10_ PFU of Sof-16. High-level viremia was registered in all infected monkeys. For both strains the level of viremia was not dependent on the inoculated dose (5 versus 6 log_10_ PFU). However, monkeys infected with Sof-16 had more delayed viremia (3^rd^–6^th^ d.p.i.) with a lower-pick than monkeys inoculated with Abs-18 (1^st^–4^th^ d.p.i.) (Figure 3A). Internal body temperature of all animals was checked daily. We registered a fever in all *M. fascicularis* infected with either strains of TBEV that correlated with the dynamics of viremia in serum. An example of the correlation for 2 infected monkeys is shown in Figure 3B and 3C.

The visceral organs and CNS of all monkeys s/c infected with both strains of TBEV were investigated for the presence of infectious virus at the early terms after infection (8–10 days) (Table 5). Virus was registered in the CNS in 4 out of 6 monkeys. Infection with 5.4 log_10_ PFU of Abs-18 resulted in the presence of virus at these terms in all investigated CNS parts. After inoculation with a higher dose of Abs-18 (6.4 log_10_ PFU) virus was registered only in one spot of the CNS (thoracic part of a spinal cord) in low titer (1.5 log_10_ PFU/ml). Among 3 monkeys infected with Sof-16 on 8–9 d.p.i. virus was detected only in the CNS of one monkey inoculated with a higher dose (6.1 log_10_ PFU). This corresponds with the late-term viremia in all monkeys inoculated with Sof-16 in comparison with Abs-18. Infectious virus was detected in the spleen of all animals and in lymph nodes and kidneys of some animals (Table 5).

**Table 5:** Virus titers in visceral organs and CNS (log_10_ PFU/ml) of *M. fascicularis* monkeys s/c inoculated with TBEV Abs-18 or Sof-16 strains at the early terms after infection (8–10 d.p.i.).

Virus	Abs-18			Sof-16		
Monkey ID	#33	#29	#28	#31	#25	#30
Viral dose in inoculum (log_10_ PFU/ml)	***6.4***	***5.4***	***5.4***	***6.1***	***5.1***	***5.1***
Day of autopsy	*10*	*8*	*10*	*9*	9	*8*
	**Viral titers in CNS**					
Frontal lobe	0	0	**2.2**	**1.7**	0	0
Cerebellum	0	**1.8**	**1.5**	0	0	0
Thoracic part of s.c.	**1.5**	**1.7**	**1.7**	**2.2**	0	0
Caudal part of s.c.	0	**2**	**1.7**	0	0	0
	**Viral titers in visceral organs**					
Spleen	**3.2**	**5.1**	**3.6**	**2.6**	**4.3**	**2.8**
Liver	0	0	0	0	0	0
Kidneys	0	**2.3**	0	0	0	0
Axillary l.n.	0	**3.2**	0	0	0	0
Inguinal l.n.	0	**4.6**	0	**1.7**	0	0
Submaxillary l.n.	0	0	2.6	0	0	0

0–<1 PFU in 0.2 ml of 10% suspension; s.c. – spinal cord, l.n. – lymph nodes.

### 5. Comparison of viremia levels in two different monkey species – *M. fascicularis* and *C. aethiops* after s/c infection with the Abs-18 strain of TBEV

A moderate to high viremia level was shown in *M. fascicularis* after s/c infection with TBEV; however, in those experiments we used cloned viruses. In order to determine whether the monkey species or the properties of the virus is more important for the expressed viremia, we decided to compare two species of monkeys using the same cloned virus, Abs-18, in the same experiment. Euthanasia of infected animals was performed at the late terms after infection, as it was done in the first experiment in *C. aethiops* monkeys with non-cloned Absettarov strain of TBEV.

Three *M. fascicularis* monkeys (#26, 27 and 34) and three *C. aethiops* monkeys (#35, 36 and 37) were s/c inoculated with 6.4 log_10_ PFU of Abs-18. Viremia was evaluated in sera on days 1–9 and 14 after infection (Figure 3D). High-level viremia was detected in all three *M. fascicularis* monkeys on days 1–4. The peak of viremia (3.1–3.9 log_10_ PFU/ml) was reached on days 3–4. In contrast, no detectable viremia was registered in all three *C. aethiops* monkeys infected with the same dose of virus (Figure 3D).

No visible clinical signs were registered in any monkey of both species infected s/c with TBEV. Nonetheless, we registered a fever in all *M. fascicularis* infected with TBEV that correlated with dynamics of viremia in a serum.

The visceral organs (lymph nodes, thymus, spleen, liver and kidneys) and CNS of all monkeys infected with Abs-18 were tested for the presence of infectious virus by plaque assay. In contrast to the previous experiment, where the high titers of the infectious virus were detected in all inspected organs at the early terms after infection (8–10 days), no virus was detected in visceral organs of any infected monkey on 22, 27 and 28 d.p.i. Only one *C. aethiops* monkey (#37) had 2.6 log_10_ PFU/ml of virus in kidneys on day 27. No infectious virus was detected in the CNS of any monkey.

### 6. Histological evaluation of CNS and visceral organs of monkeys s/c infected with TBE virus

**Figure 4:**
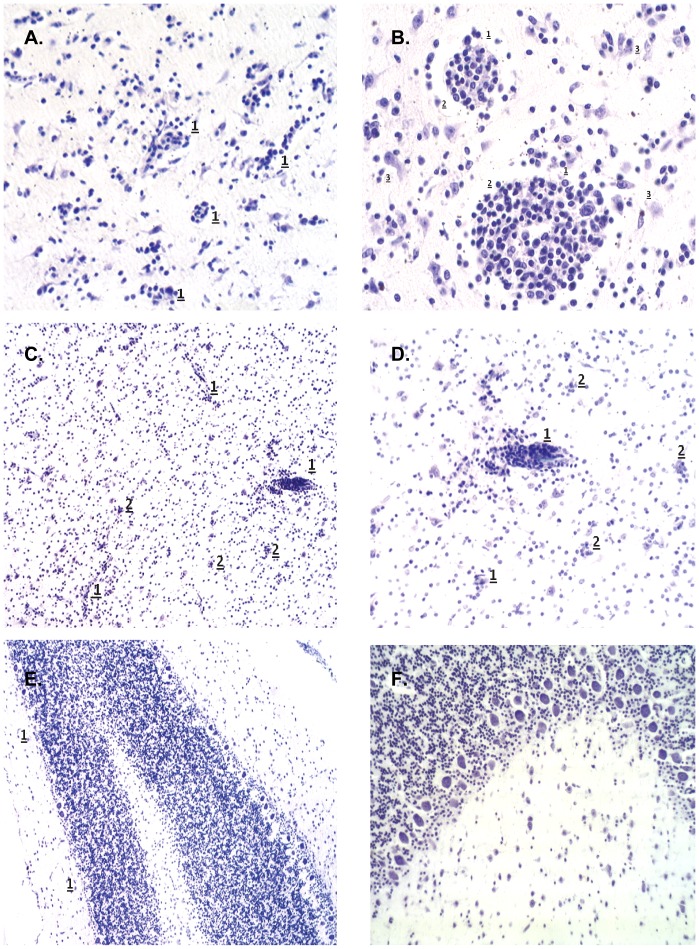
Histological lesions in the brain of monkeys infected with 6.0 log_10_ PFU of Abs-18 at the late terms after infection (27–28 days). A and B. Brainstem (*truncus cerebri*) of *M. fascicularis* monkey #26: multiple small vasculitis (1), perivascular edema (2), degenerative changes in neurons (3). Magnification: (A) ×100, (B) ×200. C and D. Subcortical region of *M. fascicularis* monkey #26: small vasculitis (1) and nodules of neuronophagia (2). Magnification: (C) ×100, (D) ×400. Cortex of cerebellum: E. Fall out of small groups of Purkinje cells in *M. fascicularis* monkey #34 (1). Magnification ×100.; F. Non-infected normal control. Magnification ×200. Staining by Nissle method was used.

**Figure 5:**
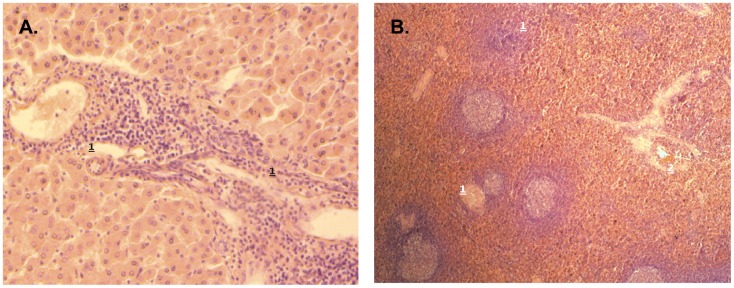
Histological lesions in the liver and spleen of *M. fascicularis* monkey (#26) at the late terms after infection with 6.0 log_10_ PFU of Abs-18. A. Liver: lymphohistiocytic infiltration of portal liver tracts (1). Magnification ×200. B. Spleen: reduction of lymphoid follicles (1); depletion of white pulp along the trabecular arteries (2). Magnification ×400. Staining with hematoxylin and eosin was used.

All monkeys of both species s/c infected with two different cloned strains of TBEV and euthanized in different terms after infection were studied for the presence of PM lesions in the CNS (in cortex, subcortical nuclei, thalamus, Ammon's Horn, midbrain, pons, Rhomboid fossa, cerebellum, medulla, cervical and lumbar spinal cord) and visceral organs. No serious lesions were found in the CNS of any monkey s/c infected with TBEV. However, slight neurological changes were found in two *M. fascicularis* monkeys (#26 and 34) at the late terms after infection (27–28 days) with 6.0 log_10_ PFU of Abs-18. The results are shown in Figure 4. Monkey #26 showed the presence of multiple small vasculitis, microglial activation and multiple small foci of infiltrative-productive reaction in the brain (Figure 4: A–D). In addition, slight histological lesions in the liver and spleen were also found in this monkey. Changes were expressed in moderate lymphohistiocytic infiltration of portal liver tracts and moderate exhaustion of white pulpa of spleen (Figure 5: A–B). Monkey #34 showed the degeneration of Purkinje cells in the cerebellum (Figure 4: E–F). Slight changes in lymph nodes and spleen were also registered in *M. fascicularis* monkey #25, infected with 5.1 log_10_ PFU of Sof-16, euthanized on day 9 after infection.

## Discussion

Development of the preventive and therapeutic antiviral preparations critically depends on the employment of an adequate model for the evaluation of their efficacy. Initially the efficacy of such preparations is estimated based on the ability to prevent acute infection, to reduce the level of animal death in experiments or to at least mitigate their clinical signs. For these purposes a mouse model is routinely used, because mice are highly susceptible to peripheral inoculation with TBEV. However, the forms of TBE that can progress after inoculation of sublethal and subclinical viral doses are difficult to model in mice, because peripheral inoculation of mice with 1 PFU of a highly virulent TBEV strain can be sufficient for the following death [18]. Although less virulent strains of TBEV for this model can be found, the efficacy of antiviral preparations sometimes have to be estimated in the model, closer to human, by its sensitivity. As different ways of immune response activation may be involved during lethal and sublethal infections, monkey models have become the most attractive.

In our experiments we attempted to apply a model for TBE and OHF after s/c inoculation of *C. aethiops* monkeys and to estimate the efficacy of TBE inactivated vaccine against both viruses in this model. *C. aethiops* monkeys have been previously used to reproduce an acute TBEV infection after s/c, intravenous or intranasal inoculation [27], [47], and for the estimation of immunogenicity of inactivated TBE vaccine [48].

We have not observed any visible clinical signs in *C. aethiops* monkeys after infection with the TBEV Absettarov strain; only a short fever was registered in 3 monkeys and none in the animals infected with its clone Abs-18. We have observed slight PM lesions in the CNS in 1 (#13) out of 7 infected African Green monkeys, euthanized more than 20 days after infection. At these terms infectious virus was revealed in the brain of only one (#22) out of 7 monkeys, infected with the Absettarov strain. However, virus was revealed in lymph nodes and spleen of all monkeys infected with Absettarov TBEV strain and in none of the animals infected with its clone. Comparison of these data allows speculation that the Absettarov strain appears to be slightly more virulent than its clone Abs-18. More over, the decreased virulence in mice after i/c and intraperitoneal inoculation was shown for Abs-18 compare to the parental Absettarov strain [40].

Thus, s/c inoculation with TBEV in *C. aethiops* monkeys results in asymptomatic or slight feverish disease that is irregularly accompanied with a subtle viremia and viral reproduction in organs of the immune system. Rare cases of infectious virus detection and PM lesions in the brain indicate that virus is able to invade the CNS after s/c inoculation in these animals. In general, the disease picture in this species of monkeys is very close to the light feverish form of TBE in patients.

Despite the obliterated form of infection in this model, we were able to show effectiveness of inactivated vaccine against TBE (“FSME-Immune”). This was indicated by neutralizing AB titers against TBEV after double vaccination, expressed AB booster already on the 1^st^ day after challenge and the absence of virus in the spleen and lymph nodes of immunized animals after challenge. However, other parameters can not be used as reliable markers in *C. aethiops* monkeys, because of their inconsistency in non-vaccinated animals.

S/c inoculation of OHFV in African Green monkeys resulted in the disease, lacking expressed clinical symptoms. Nevertheless, monkeys infected with the virus had a massive hemolytic syndrome and thrombocytopenic purpura, against the background of viremia, virus reproduction in the CNS, visceral and immune system organs. A clinical presentation with similar markers is often observed in patients with OHF. Double immunization of *C. aethiops* monkeys with inactivated TBE vaccine (produced in EIPVE) led to the expressed immune response with high neutralizing AB titers against TBE and OHF viruses with a booster immediately after challenge. Protective effect of the vaccine was expressed in the absence of hemolytic syndrome in vaccinated monkeys challenged with OHFV. In this case even a simple complete blood count was very informative. However, based on the presence of virus in the CNS and visceral organs, we can conclude that vaccination did not prevent the infection or viral spread and reproduction in visceral organs, immune system and CNS. It is important that after immunization with inactivated TBE vaccine high neutralizing AB titers against OHFV were observed. Thus, the data on immunogenicity of the vaccine are not sufficient for a conclusion about its protective properties, especially in the case of protection against heterologous virus.

In the absence of visible clinical representation during TBE infection the most important markers are: the presence and severity of PM lesions and virus titers in the CNS. Other markers may include the level of viremia, virus reproduction in peripheral organs and the duration of virus persistence that can serve as evidence of chronic infection. The level of viremia is especially important, because it is vital and allows estimating the probability of virus penetration into CNS. It also reflects the chance of appearance of immune-escape mutants' and antiviral drug-resistant mutants' in a viral population.

In this regard, *C. aethiops* is not the best model for TBEV infection. In contrast, s/c inoculation of *M. fascicularis* monkeys with two different TBEV strains led to a febrile disease with a fever, correlating with dynamics of serum viremia, very well expressed viremia, active virus reproduction in spleen and virus penetration into the CNS. The distribution of infectious virus in peripheral organs and CNS was dependent on the TBEV strain. Abs-18 appeared to be slightly more informative and prospective for the estimation of efficacy of preventive preparations than Sof-16 in *M. fascicularis* model. In addition, better sensitivity to TBEV of macaques compare to African Green monkeys was demonstrated during simultaneous infection of two species of monkeys with the same dose of Abs-18 virus. In addition, slight neurological changes were registered in two *M. fascicularis* monkeys at the days 27–28 after infection in this comparative experiment. Thus, monkeys of this species can be successfully used for the modeling of the most frequent form of TBE in humans.

No infectious virus was detected in the CNS or spleen of *C. aethiops* monkeys (#11 and #13) at the late terms (day 48) after infection with TBEV. However, virus penetration into the CNS and its detection in a spleen at the terms following 20 d.p.i. suggest that the proposed monkey model can be used to study chronic forms of TBE, as it has been shown on *M. mulatta* (Frolova and Pogodina, 1984). Additional experiments with different terms and with other TBEV strains and/or doses may help to further explore the usefulness of this model. Despite the small number of animals used, we noticed that higher dose of inoculated virus resulted in faster elimination from the organism. This can be tracked by the persistence of viremia and the titers of virus in the visceral organs and in CNS. It also has been noticed in earlier studies in *Macaca mulatta* with Langat virus [36].

The macaque model described in our current work allows estimating the possibility of the vaccine candidates to prevent virus penetration into the CNS. The optimal terms for estimation of the viral titers in CNS are 8–16 d.p.i. After day 20 the infectious virus in CNS was registered only in some animals. Similar data were obtained after infection of *C. aethiops* with OHFV. Virus persistence can be estimated at more than 30 days after infection by the testing of CNS for the presence of infectious virus or viral RNA.

Overall, two models of s/c infection of monkeys with two different tick-borne Flaviviruses, which is close to natural virus transmission in humans, were explored here. S/c inoculation of *C. aethiops* monkeys with TBEV results in slight feverish disease accompanied with a subtle viremia and viral reproduction in organs of the immune system. In this model the level of protection can be assessed only postmortem by the absence of virus in the spleen, lymph nodes and CNS of immunized animals after challenge. In contrast, infection of *M. fascicularis* with TBEV led to a febrile disease with well expressed viremia, a fever, correlating with dynamics of viremia in a serum, and active virus reproduction in visceral organs. In both monkeys' species virus was able to invade the CNS after s/c inoculation. The protection level of future vaccine candidates against OHFV can be estimated in *C. aethiops* by the absence of hemolytic syndrome in vaccinated monkeys, as well as by the absence of virus in the CNS and visceral organs.
